# Urgent Spinal Surgery in a Lateral Decubitus on a Patient with a Left Ventricular Assist Device on Full Anticoagulation: A Case Report

**DOI:** 10.7759/cureus.55266

**Published:** 2024-02-29

**Authors:** Angelique S Do, Monis A Khan, Lindsey Ross, Robert Ravinsky, Adam J Milam, Seung J Lee, Omar Durra, J. Patrick Johnson

**Affiliations:** 1 Department of Neurosurgery, Cedars-Sinai Medical Center, Los Angeles, USA; 2 Department of Neurologic and Orthopedic Surgery, University of Arizona, Phoenix, USA; 3 Department of Orthopaedics and Physical Medicine, Medical University of South Carolina, Charleston, USA; 4 Department of Anesthesiology, Cedars-Sinai Medical Center, Los Angeles, USA; 5 Department of Neurosurgery, Mayo Clinic, Jacksonville, USA

**Keywords:** spinal surgery, left lateral decubitus position, laminotomy, microdiscectomy, lvad

## Abstract

This case report aims to demonstrate the feasibility of performing spinal surgery in patients with a left ventricular assist device (LVAD), who are traditionally considered unsuitable candidates due to the need for anticoagulation and the challenges associated with the prone position.

A case of a patient with an LVAD undergoing microdiscectomy in the left lateral decubitus position is presented. The procedure was carried out by a specialized interdisciplinary team with appropriate monitoring.

The patient underwent the procedure safely, demonstrating that spinal surgery can be performed in patients with LVAD without reversing anticoagulation or resorting to the prone position. This approach mitigates the risk of thrombotic events and hemodynamic instability.

This case study suggests that spinal surgery, specifically microdiscectomy, can be safely performed in patients with LVAD using the left lateral decubitus position. This finding has significant implications for patients who are unable to ambulate and therefore struggle to qualify for a heart transplant.

## Introduction

The heart transplant waitlist is increasing by 34% annually due to advanced heart failure and limited donor availability [1]. Left ventricular assist devices (LVADs) serve as a bridge to transplant and destination therapy for ineligible patients [2]. However, LVAD patients may require urgent surgeries, and ambulation is crucial for a better prediction of prognosis [3]. Back pain limits mobility in some patients, and there is limited data on surgical safety, especially for spinal procedures. We present the first lamino-foraminotomy in an LVAD patient, demonstrating the safety of lateral decubitus position for microdiscectomy with careful planning and a multidisciplinary approach.

## Case presentation

A 52-year-old male with American Society of Anesthesiologists (ASA) physical status IV was diagnosed with ischemic cardiomyopathy and required LVAD implantation in 2017. His medical comorbidities included diabetes, hypertension, and coronary artery disease status post-stent placement (x4) in 2008. He was placed on aspirin and warfarin for maintenance. Over the past few years, he began complaining of chronic back and left leg pain, initially managed conservatively. The pain acutely worsened, and he was admitted to the hospital with severe, incapacitating pain where he was unable to stand or ambulate for more than a few minutes and was essentially bed-bound. On neurologic examination, he had weakness in the dorsiflexors with 4/5 pain-limited strength and left leg paresthesias in an L5-S1 dermatomal distribution. A left straight leg test was positive, and he had a severely antalgic gait.

**Figure 1:**
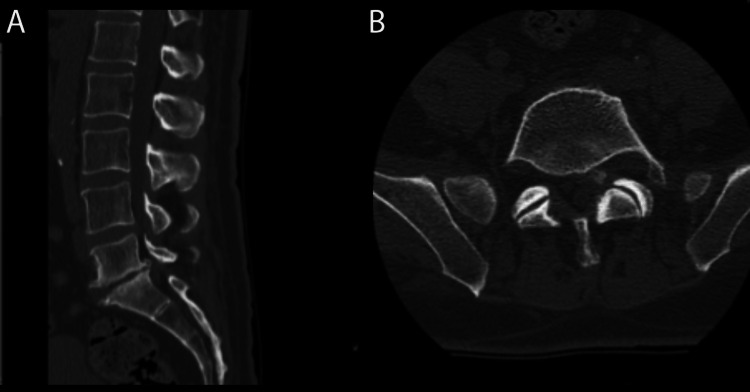
CT lumbar spine A. Sagittal view and B. Axial view exhibiting left paracentral partially calcified disc herniation

A computed tomography (CT) scan of the lumbar spine showed evidence of an L5-S1 degenerative disk with osteophytic formation and loss of disc height. There was calcification in the left L5-S1 lateral recess, suggestive of heterogenous disk herniation (Figures 1a, 1b). The plan was for laminoforaminotomy and microdiscectomy. The patient was maintained on his regular anticoagulation and antiplatelet therapy (last dose of aspirin and warfarin, the evening before surgery). Additionally, the patient underwent a right heart catheterization the day prior to surgery and the filling pressure and cardiac index were normal on LVAD at 2600 rpm.

Operation

On the day of surgery, the international normalized ratio (INR) was 2.2; the thromboelastography (TEG) study showed supranormal fibrinogen levels (maximal clot firmness of 35 mm). The patient was accompanied to the operating room by the cardiovascular anesthesia team and a cardiac perfusionist, who remained in the operating room for frequent monitoring of the LVAD. Before induction, the LVAD settings included flow 4.3 L/min, pump speed 8940 rotations per minute (RPM), and pulsatility index (PI) 6.2. The patient's vital signs prior to induction of anesthesia were noted as a systolic blood pressure of 99 mmHg, a diastolic blood pressure of 77 mmHg, a heart rate ranging between 60 and 85 beats per minute, oxygen saturation of 100%, a respiratory rate of 10 breaths per minute, and a body temperature of 98.4 degrees Fahrenheit. The patient underwent general anesthesia with endotracheal intubation. Pre-induction, a radial arterial line was placed for hemodynamic monitoring. A transesophageal echocardiography (TEE) probe was inserted into the esophagus for hemodynamic monitoring. He was induced with a regimen that included 50 mg of propofol, 25 mcg of fentanyl, and 50 mg of rocuronium. For maintenance of anesthesia, sevoflurane was given at a concentration of 0.9% to 1.1%. To maintain the patient's systolic blood pressure within the desired range of 100 to 130 mmHg throughout the surgery, a continuous infusion of norepinephrine at 4 mcg per minute was administered. This vasopressor was promptly decreased and discontinued after the patient was extubated.

**Figure 2:**
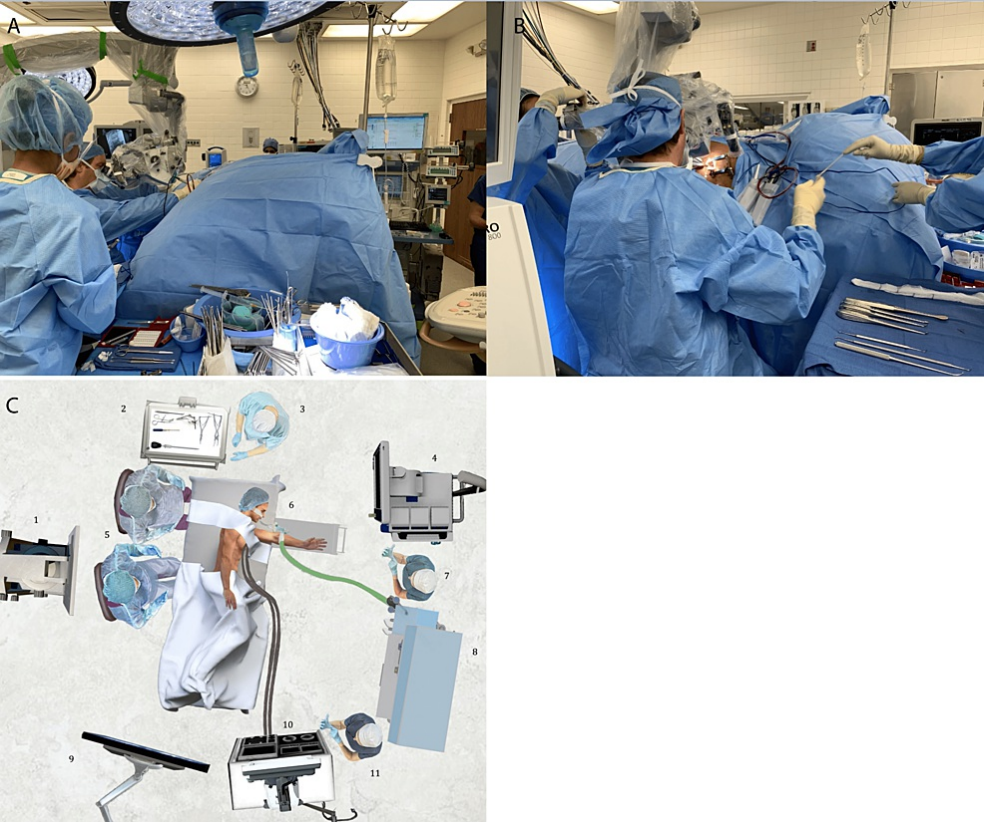
Intraoperative setup with the patient in the left lateral decubitus position A. The surgeon is seated with the patient facing anesthesia. B. The assistant is to the left and the surgical technician to the right of the surgeon. C. Diagram of the operating theater.

Defibrillator pads were placed with the patient in the right lateral decubitus position. His cardiac parameters were carefully monitored to ensure hemodynamic stability during positioning. His lumbar region was prepped and draped in a standard fashion and a midline incision was made. The exposure was extended down into the lamina at the L5-S1 level and the operative microscope was introduced into the field. The surgical team was seated to allow for improved access at the lateral patient position (Figure 2). A left L5 and S1 hemilaminectomy, medial facetectomy, and foraminotomy were completed and multiple large fragments of degenerative and calcified disk material were removed. The traversing S1 nerve root and the exiting L5 nerve were thoroughly decompressed. Hemostasis was achieved with Avitene, Gelfoam, bone wax, and bipolar cautery. The wound was closed and a deep fascial drain was left in place. The estimated blood loss was 200 mL. The patient was extubated and transferred to the post-anesthesia care unit (PACU) in stable condition. His immediate postoperative international normalized ratio (INR) was at 2.2.

Clinical course

**Figure 3:**
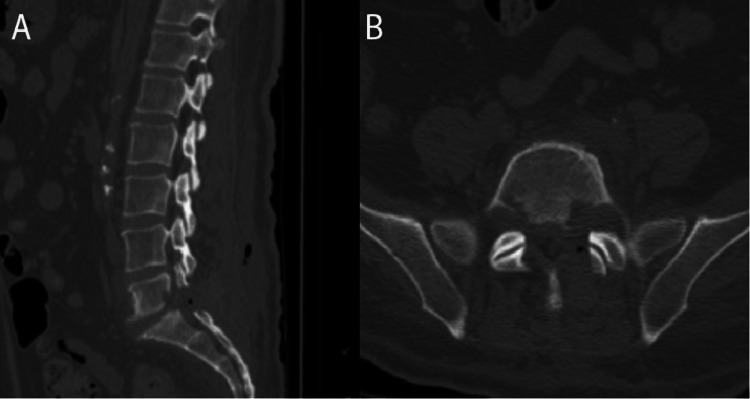
Postoperative CT lumbar spine in A. Sagittal view and B. Axial view, exhibiting interval removal of L5-S1 lamina, medial facet, calcified disc herniation, and part of the L5 vertebral body

Postoperatively, the patient’s ability to stand and ambulate improved. He had full strength except for the left extensor hallucis longus (EHL), which was 4/5 and his paresthesias moderately persisted. The surgical pathological evaluation exhibited focal acute inflammation of the soft tissue and bone with areas of gram-positive staining suspicious for acute osteomyelitis with associated osteonecrosis. Thus, vancomycin was started, and the patient underwent a CT-guided L5-S1 biopsy (Figure 3). The Gram stain and subsequent biopsy cultures were negative on postoperative day 9. The Infectious Disease consultant recommended peripheral intravenous daptomycin for six weeks. At the patient’s two-month follow-up appointment, he no longer required opioids and was ambulating without assistance. He continued to complain of minimal numbness in his left leg. Approximately three months after his microdiscectomy, he received an orthotopic heart transplant and is doing well.

## Discussion

This case report describes a patient with an LVAD for a bridge-to-heart transplant, undergoing an L5-S1 microdiscectomy for disc herniation and degeneration. The number of patients with ventricular assist devices in this country is continuing to increase [4].

This case highlights several surgical and anesthetic considerations, including hematologic concerns, patient positioning, and operating room set-up for spine surgery in LVAD patients.

Hematologic considerations

LVAD patients receive anticoagulation and/or antiplatelet therapy to prevent thromboembolic complications [5]. Several studies incorporate various anticoagulation reversal protocols for LVAD patients, although they mostly report great outcomes from emergent and urgent non-cardiac procedures (Table 1) [6]. Garatti et al. studied 11 LVAD patients undergoing noncardiac surgeries, reporting no major complications or mortalities including, a patient who underwent craniotomy for intracranial hematoma evacuation [7]. Similarly, Morgan and colleagues examined complications in 25 non-cardiac surgeries among patients with LVADs, there were bleeding complications in patients on both warfarin and aspirin preoperatively, and the incidence of bleeding was associated with preoperative INR [6]. As with other studies, there were no major complications noted.

**Table 1:** LVAD patient's elective and neurosurgical procedures, operative interventions, morbidities, and mortalities LVAD = left ventricular assist device; NA = not available

Elective + LVAD	Year published	# of patients	# of surgeries	# used intraop inotropic support	# used intraop-invasive hemodynamic monitoring	# of patients requiring blood products	Major postoperative complications	Mortality
Goldstein et al. [8]	1995	8	12	1	2	4	4 hypotension with positional change, 1 hypotension, 1 hemorrhage	0
Ahmed et al. [9]	2012	6	6	0	1	0	0	0
Bhat et al. [10]	2012	27	52	NA	NA	NA	NA	0
Morgan et al. [6]	2012	19	22	NA	NA	8	7 hemorrhage, requiring transfusions	0
Schmid et al. [11]	2001	5	6	NA	NA	NA	3 hemorrhage, requiring exlap	NA
Arnaoutakis et al. [12]	2013	NA	38	NA	NA	NA	None	0
Garatti et al. [7]	2008	11	12	4	11	10	None	0
Neurosurgery + LVAD	Year published	Operation	# of surgeries	# used intraop inotropic support	# used intraop-invasive hemodynamic monitoring	# of patients requiring blood products	Major postoperative complications	Mortality
Schmid et al. [11]	2001	ICH causing ICP issues	1	NA	NA	NA	1 redo craniotomy for worsening ICP after the first craniotomy	Secondary to respiratory failure
Bhat et al. [10]	2012	decompressive lami	1	NA	NA	NA	NA	0
Vandse & Papadimos [13]	2015	Brain tumor	1	1	1	1	0	0
Kollmar [14]	2017	lumbar decompression	1	1	1	1	Hypotension with positional change, bleeding	0

Neurosurgical interventions in patients with LVAD have been reported in small case series, mostly for emergent cases and neuroendovascular interventions [15-17]. To our knowledge, only two urgent non-vascular neurosurgical cases in patients with LVAD have been published. Vandse and Papadimos reported on a patient who underwent a right temporal craniotomy for tumor resection [13]. The patient was found to have four ring-enhancing lesions with evidence of edema, uncal herniation, and an 8 mm midline shift on imaging. Notably, this patient had been managed with a lower INR goal of 1.3-1.8 given a history of GI bleeding. Preoperatively, the warfarin and aspirin were discontinued, and the patient’s INR was 1.6. The patient was given two units of fresh frozen plasma and two units of platelets to decrease the bleeding risk. The patient had an uneventful operative course, anticoagulation was resumed in the form of intravenous heparin drip 36 hours after surgery. In our current case, the patient had an uncomplicated intraoperative course without any changes to his anticoagulation regimen; he had an INR of 2.2 on the day of surgery and this was maintained at >2.0 postoperatively. Decisions regarding anticoagulation management and reversal should be based on indications for anticoagulation (e.g. atrial fibrillation), risk of surgical bleeding (i.e. minor versus major surgeries), and risk of thromboembolic events from the LVAD.

Patient positioning

Kollmar et al. presented an urgent case of posterior spine surgery in a patient with an LVAD [14]; during prone positioning of the patient, the patient became hypotensive with a mean arterial pressure (MAP) of 50, which was unresponsive to an initial 250 mL bolus of normal saline. Norepinephrine was required to maintain a MAP > 65 mmHg. This hypotension was attributed to hemodynamic changes from the positioning. Several studies have documented the hemodynamic changes after changing a patient to prone positioning. There is a reduction in preload, stroke volume, and cardiac index compared to the supine position; these changes are attributed to compression of the inferior vena cava and increased intrathoracic pressure [18,19]. The hemodynamic changes may be minimized with lateral positioning. Additionally, lateral positioning allows relatively easy placement of the TEE probe without the need for intraoperative repositioning and the dangers that accompany these maneuvers in the case of emergencies. TEE is used in select cases to allow for careful monitoring and assessment of the ventricular function and pressures [20].

The prone position is commonly favored due to surgeons' familiarity and comfort, but spine surgery in the lateral position has been reported as safe and effective with several advantages [21]. These include reduced bleeding due to decreased abdominal pressure and epidural venous engorgement and fewer hemodynamic changes compared to the prone position, making it advantageous for obese and elderly patients. Additionally, the lateral position allows for both anterior and posterior surgeries without the need for repositioning, and adjustments in hip and knee positions can modify kyphosis and lordosis.

Particularly in the lumbar region, there was a concern that the lateral decubitus position would result in smaller lumbar interspinous distances, but Agarwal et al. noted similar interspinous distances when comparing the modified lateral decubitus position versus the prone position [21]. Here, the modified lateral decubitus position was defined as the knee-to-chest position. Another group in China, Du et al. noted that even in cervical laminoplasties, the lateral position had advantages over the prone position, especially in obese patients [22].

Cardiac anesthesia considerations

There are several anesthetic considerations in this patient population centered around a multidisciplinary approach to the preoperative workup. Patients with LVADs are closely followed by a mechanical circulatory support (MCS) service, which generally includes a cardiologist, nurses, and social workers. In this case, the anesthesiologist was notified about a week before the surgery in order to consult with the appropriate specialties. The patient had a transthoracic echocardiogram a week prior to the surgery; patients with LVAD generally undergo routine surveillance to evaluate for changes in native heart function and valvular abnormalities [23]. Additional preoperative workup may include LVAD interrogation and right heart catheterization, both of which are especially important for major surgeries. The anesthesiologist should also discuss positioning and management of anticoagulation with the MCS and surgical team, which help with decisions regarding blood products and reversal agents (see prior sections).

Monitoring of LVAD patients undergoing elective non-cardiac surgery has been discussed elsewhere [9,11] but briefly, the literature continues to support the availability of a TEE probe to monitor fluid status (i.e. left-sided filling pressures), a perfusionist positioned in the operating room for the entirety of the procedure to manage the LVAD, continuous invasive arterial pressure monitoring to monitor hemodynamics (as non-invasive may be inefficient in non-pulsatile patients), and central access for administration and fluids, blood products, and vasoactive drugs [24]. The major anesthetic consideration for this case is the use of a multidisciplinary team that decides on anticoagulation, monitoring, positioning, and OR set-up.

## Conclusions

We present the first case of an elective microdiscectomy on a patient with an LVAD who did not have any modifications to his anticoagulation and antiplatelet therapy. With the appropriate perioperative INR goals, the balance of the risk of coagulopathy in the setting of LVAD versus the risk of uncontrolled intraoperative bleeding can be optimized. Patient positioning must also be planned carefully and monitored for any hemodynamic derangements. Here, we demonstrate the safety of lateral decubitus positioning for microdiscectomy. Multidisciplinary teamwork ensures safe elective neurosurgical intervention for LVAD patients on anticoagulation and antiplatelet therapy.
